# *PbLAC4-like*, activated by *PbMYB26*, related to the degradation of anthocyanin during color fading in pear

**DOI:** 10.1186/s12870-021-03220-1

**Published:** 2021-10-13

**Authors:** Guangping Zhao, Fangxin Xiang, Shichao Zhang, Junxing Song, Xieyu Li, Linyan Song, Rui Zhai, Chengquan Yang, Zhigang Wang, Fengwang Ma, Lingfei Xu

**Affiliations:** 1 College of Horticulture, Northwest A&F University, Taicheng Road NO.3, Yangling, Shaanxi Province China; 2 State Key Laboratory of Crop Stress Biology for Arid Areas, Northwest A&F University, Taicheng Road NO.3, Yangling, Shaanxi Province China

**Keywords:** Anthocyanin degradation, *PbLAC4-like*, *PbMYB26*, Pear

## Abstract

**Background:**

Decrease in anthocyanin content results in the loss of red color in leaves, petals and receptacles during development. The content of anthocyanin was affected by the biosynthesis and degradation of anthocyanin. Compared with the known detailed mechanism of anthocyanin biosynthesis, the degradation mechanism is not fully investigated. It is vital to study the degradation mechanism of anthocyanin in pear for promoting the accumulation of anthocyanin and inhibiting the red fading in pear.

**Results:**

Here, we reported that laccase encoded by *PbLAC4-like* was associated with anthocyanin degradation in pear. The expression pattern of *PbLAC4-like* was negatively correlated with the content of anthocyanin during the color fading process of pear leaves, petals and receptacles. Phylogenetic analysis and sequence alignment revealed that *PbLAC4-like* played a vital role in anthocyanin degradation. Thus, the degradation of anthocyanin induced by *PbLAC4-like* was further verified by transient assays and prokaryotic expression. More than 80% of anthocyanin compounds were degraded by transiently over-expressed *PbLAC4-like* in pear fruitlet peel. The activity of crude enzyme to degrade anthocyanin in leaves at different stages was basically consistent with the expression of *PbLAC4-like.* The anthocyanin degradation ability of prokaryotic induced PbLAC4-like protein was also verified by enzyme activity assay. Besides, we also identified *PbMYB26* as a positive regulator of *PbLAC4-like.* Yeast one-hybrid and dual luciferase assay results showed that *PbMYB26* activated *PbLAC4-like* expression by directly binding to the *PbLAC4-like* promoter.

**Conclusions:**

Taken together, the *PbLAC4-like* activated by *PbMYB26,* was involved in the degradation of anthocyanin, resulting in the redness fading in different pear tissues.

**Supplementary Information:**

The online version contains supplementary material available at 10.1186/s12870-021-03220-1.

## Background

Anthocyanins are formed by glycosylation of anthocyanidins and glycosides and have a basic C6-C3-C6 skeleton [1, 2]. Anthocyanins are significant pigments that make plant tissues appear red, purple, blue, and black [3–5], so they can attract pollinators and seed carriers, thus expanding the area and scope of plant distribution [6]. Anthocyanins respond to various biotic and abiotic stresses to improve plant resistance and make plants better adapt to the environment [7]. Besides, anthocyanins are helpful for people to fight against diseases [8–10]. However, during the development process of some pear leaves, petals, receptacles, the anthocyanin content is reduced, resulting in the loss of color. The content of anthocyanin is affected not only by the anthocyanin biosynthesis but also by the anthocyanin degradation. Compared with the detailed study on anthocyanin biosynthesis, the knowledge of anthocyanin degradation is not clear enough.

In plants, the biosynthesis of anthocyanin is catalyzed by a series of enzymes, mainly including phenylalanine ammonia-lyase, chalcone synthase, chalcone isomerase, flavone 3-hydroxylase, dihydroflavonol 4-reductase, anthocyanidin synthase/leucoanthocyanidin dioxygenase, and UDP glucose: flavonoid 3-O-glucosyl transferase (UFGT). And the expression of related structural genes is synergistically regulated by the MBW (MYB-bHLH-WD40) transcription complex [11, 12]. In pear, UFGT was identified as a key enzyme involved in anthocyanin biosynthesis [13]. *MYB10*, *MYB10b*, *ERF22*, *REVEILLE* promoted the biosynthesis of anthocyanin by activating the expression of anthocyanin biosynthesis genes in pear [14–16]. *COP1.1* and *MYB120* were identified as inhibitory regulators of anthocyanin biosynthesis in pear [17, 18]. *bHLH3*, *MYB88*, *MYB124*, *NAC52* and in apple [19–21], *MYB75*, *MYB90*, *MYB113 and MYB114* in *Arabidopsis thaliana* [22–24] also were shown to promote anthocyanin biosynthesis. *MYB6* in apple [25] and *MYB1* in *Gerbera hybrid* [26] were identified to inhibit anthocyanin biosynthesis.

At present, there have been some studies on the degradation mechanism of anthocyanin in plants. Anthocyanin degradation was associated with a variety of enzymes, including peroxidases (POD), polyphenol oxidases (PPO), β-glucosidases and laccases (LAC). During the browning of *Litchi* fruit pericarp, LAC degraded anthocyanin by the model of anthocyanin-LAC-epicatechin [27]. The browning of *Litchi* fruit pericarp was also related to POD and PPO [28–30]. The flower color change from dark purple to pure white was caused by POD (BcPrx01) and β-glycosidase (BcXyl) in *Brunfelsia calycina* [31–33]. Transcriptional analysis revealed the color fading process of ‘Red Bartlett’ might be related to POD and LAC [34]. The β-glucosidase was purified from blood orange juice and it was found that β-glucosidase was closely related to the degradation of anthocyanin in pericarp and juice [35].

LACs are copper-containing polyphenol oxidases that use molecular oxygen to oxidize various aromatic and non-aromatic compounds [36]. So far, the function of LAC was mostly related to lignin accumulation. LAC played a role in the formation of lignin by promoting oxidative conjugated monolignols in sycamore maple [37, 38]. In *Arabidopsis* and poplar, lignin content decreased when *LAC* genes were RNA interference [39–42]. Some studies have also shown that the *LAC* genes in pear were closely related to the formation of stone cells caused by lignin accumulation [43, 44]. Only a few studies have shown that LAC was associated with anthocyanin degradation. The degradation of anthocyanin by LAC resulted in the browning of *Litchi* fruit pericarp [27]. Many regulators of *LAC* have been identified, but all of them were related to lignin accumulation. *MYB26*, *MYB46* and *MYB83* in *Arabidopsis* [45–48], *miR397a* and *MYB169* in pear [43, 44] were shown to involve in lignin accumulation by regulating *LAC*.

The leaves, petals and receptacles of some pear species lost color during development. According to the previous data on the differences between ‘Zaosu’ and ‘Red Zaosu’ leaves [49], we speculated *PbLAC4-like* probably related to the color fading of pear. In this study, there was a negative correlation between *PbLAC4-like* expression level and anthocyanin content during the color fading of pear leaves, petals and receptacles. Transiently overexpressed *PbLAC4-like* in pear fruitlet peel and the enzyme activity test for the degradation of anthocyanin further proved the role of *PbLAC4-like* in the degradation of anthocyanin. The regulator of *PbLAC4-like* has also been preliminarily analyzed and found that *PbMYB26* could directly bind to the promoter of *PbLAC4-like* to up-regulate its expression. These results showed that *PbLAC4-like*, activated by *PbMYB26*, promoted the degradation of anthocyanin in pear. This provided a theoretical reference for regulating the degradation of anthocyanin.

## Results

### Anthocyanin content and *PbLAC4-like* expression level during the color fading process

**Fig. 1:**
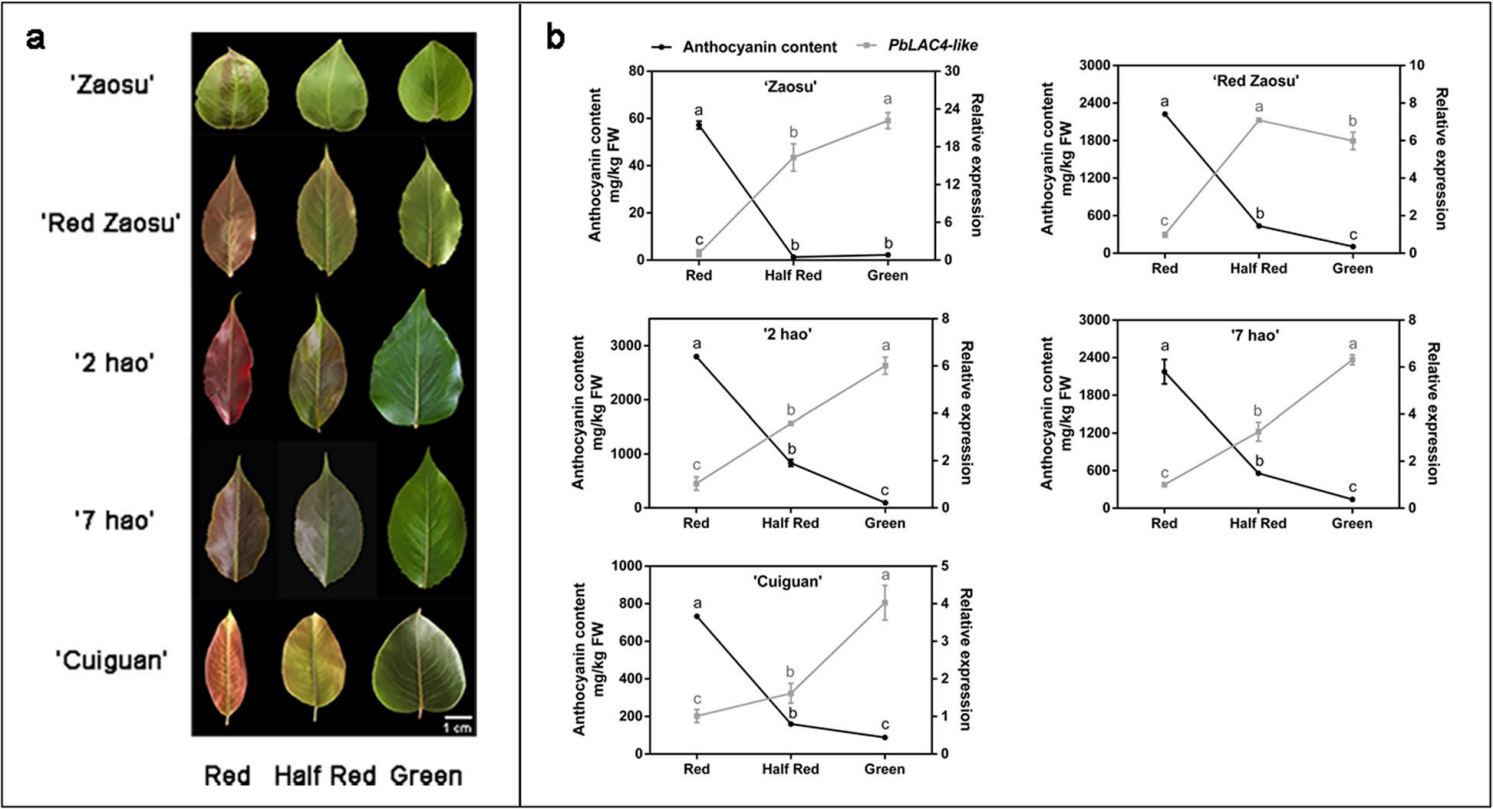
Phenotypes, anthocyanin content and *PbLAC4-like* expression level in pear leaves at different stages. **a** The phenotypes of pear leaves. **b** Anthocyanin content and the expression level of *PbLAC4-like* gene in leaves of various varieties. The significant difference was determined by Tukey test. Error bars represent the means ± SEM of three biological replicates

**Fig. 2:**
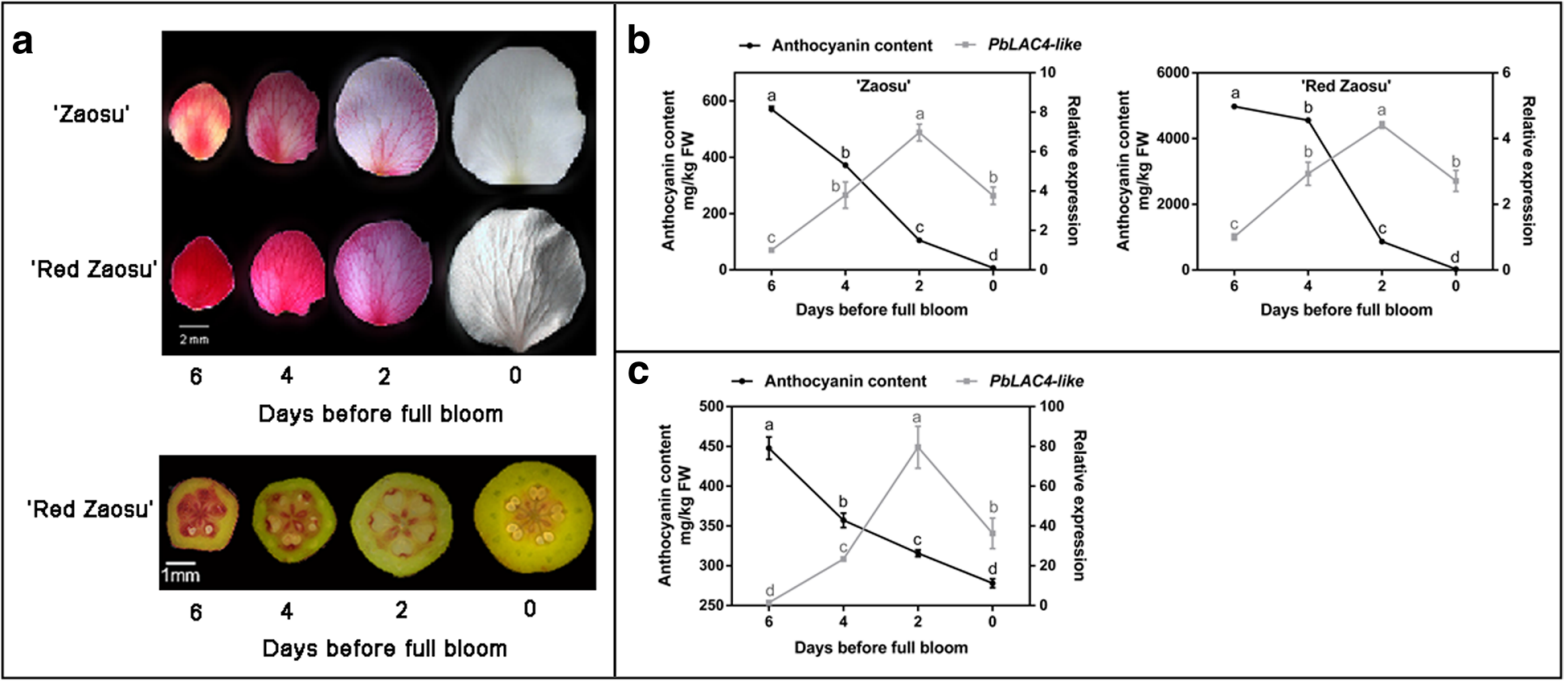
Phenotypes, anthocyanin content and *PbLAC4-like* expression level in pear petals, receptacles at different stages. **a** The phenotypes of pear petals, receptacles during color fading. **b** Anthocyanin content and the expression level of *PbLAC4-like* gene in petals of ‘Zaosu’ and ‘Red Zaosu’ at four stages. **c** Anthocyanin content and the expression level of *PbLAC4-like* gene in receptacles of ‘Red Zaosu’ at four stages. The significant difference was determined by Tukey test. Error bars represent the means ± SEM of three biological replicates

Some pear leaves, petals, receptacles exist color fading phenomenon during development (Fig. 1a). To study the relationship between color fading and *PbLAC4-like*, anthocyanin content and *PbLAC4-like* expression level in leaves of five pear varieties (‘Zaosu’, ‘Red Zaosu’, ‘2 hao’, ‘7 hao’ and ‘Cuiguan’) at three development stages (Red, Half Red, Green) were determined. During the color fading of leaves, the content of anthocyanin gradually decreased. The *PbLAC4-like* gene expression level was higher during Green and Half Red than that in Red (Fig. 1b). A correlation analysis revealed a negative correlation between anthocyanin content and *PbLAC4-like* expression level in leaves (Table 1). To further investigate the relationship between *PbLAC4-like* expression level and anthocyanin content, the *PbLAC4-like* expression level and anthocyanin content in ‘Zaosu’, ‘Red Zaosu’ petals and ‘Red Zaosu’ receptacles at 6 days, 4 days, 2 days and 0 days before full bloom were determined, respectively. In general, from 6 days before full bloom to full bloom, the *PbLAC4-like* expression level in petals and receptacles increased, while the content of anthocyanin in petals and receptacles reduced (Fig. 2b, c). Correlation analysis of *PbLAC4-like* expression level and anthocyanin content in petals and receptacles showed that they were negatively correlated (Tables 2, 3). In addition, the expression of *PbMYB10* and *PbUFGT* related to anthocyanin biosynthesis in leaves, petals and receptacles was positively correlated with the content of anthocyanin (Additional file 1: Figure S1 and Tables 1, 2, 3). In the later stage, the expression of *PbMYB10* and *PbUFGT* decreased, and the content of anthocyanin decreased, but there was still a small amount of expression. These results suggested that *PbLAC4-like* possibly played an important role in inhibiting the accumulation of anthocyanin during color fading progress.

**Table 1:** Correlation analysis of anthocyanin content and expression level of anthocyanin-related genes in leaves

	Anthocyanin				
	‘Zaosu’	‘Red Zaosu’	‘2 hao’	‘7 hao’	‘Cuiguan’
*PbLAC4-like*	−0.950**	−0.946**	− 0.964**	−0.907**	− 0.714*
*PbMYB10*	0.977**	0.985**	0.978**	0.837**	0.881**
*PbUFGT*	0.982**	0.994**	0.963**	0.891**	0.918**

**Correlation is significant at the level of 0.01 (bilateral); *Correlation is significant at the 0.05 level (bilateral)

**Table 2:** Correlation analysis of anthocyanin content and expression level of anthocyanin-related genes in petals

	Anthocyanin	
	‘Zaosu’	‘Red Zaosu’
*PbLAC4-like*	−0.707*	−0.587*
*PbMYB10*	0.663*	0.674*
*PbUFGT*	0.706*	0.768**

*Correlation is significant at the 0.05 level (bilateral); **Correlation is significant at the level of 0.01 (bilateral)

**Table 3:** Correlation analysis of anthocyanin content and anthocyanin-related genes expression in ‘Red Zaosu’ receptacles

	Anthocyanin
*PbLAC4-like*	−0.662**
*PbMYB10*	0.951**
*PbUFGT*	0.334

**Correlation is significant at the level of 0.01 (bilateral)

### Identification and analysis of *PbLAC4-like*

**Fig. 3:**
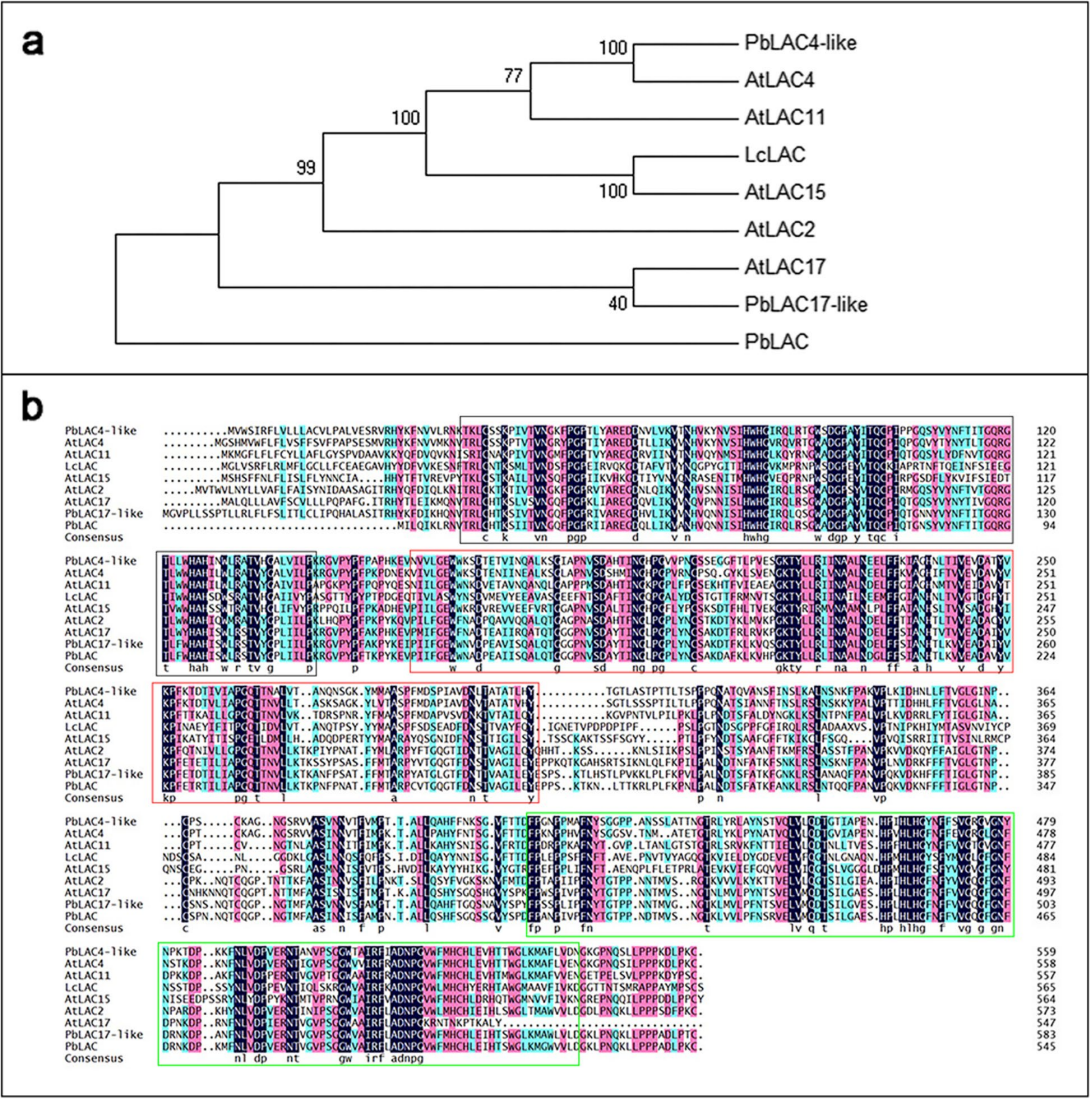
Sequence analysis of PbLAC4-like protein. **a** Phylogenetic analysis of PbLAC4-like protein and LACs from other plants. **b** Sequence alignment of PbLAC4-like protein and LACs from other plants (The black, red and green boxes represent three copper oxidation domains, respectively)

The complete coding sequence length of *PbLAC4-like* was 1680 bp, and the PbLAC4-like protein consisted of 559 amino acids. PbLAC4-like protein belonged to the LACs family, which was multicopper oxidase found in plants, fungi, and bacteria [36]. A phylogenetic tree containing PbLAC4-like protein and previously characterized LACs in plants was constructed, indicating high homologies of PbLAC4-like protein to LcLAC and AtLAC15 involved in the degradation of flavonoid [27, 50] (Fig. 3a). Besides, the protein sequence alignment results showed that the similarity of PbLAC4-like protein sequence with LcLAC and AtLAC15 was 44.72 and 41.99%, respectively, mainly concentrated in three copper oxidation domains (Fig. 3b). These results showed that the *PbLAC4-like* gene inhibited the accumulation of anthocyanin by degrading anthocyanin.

### Transient overexpression of *PbLAC4-like* gene in the peel of pear fruitlet

**Fig. 4:**
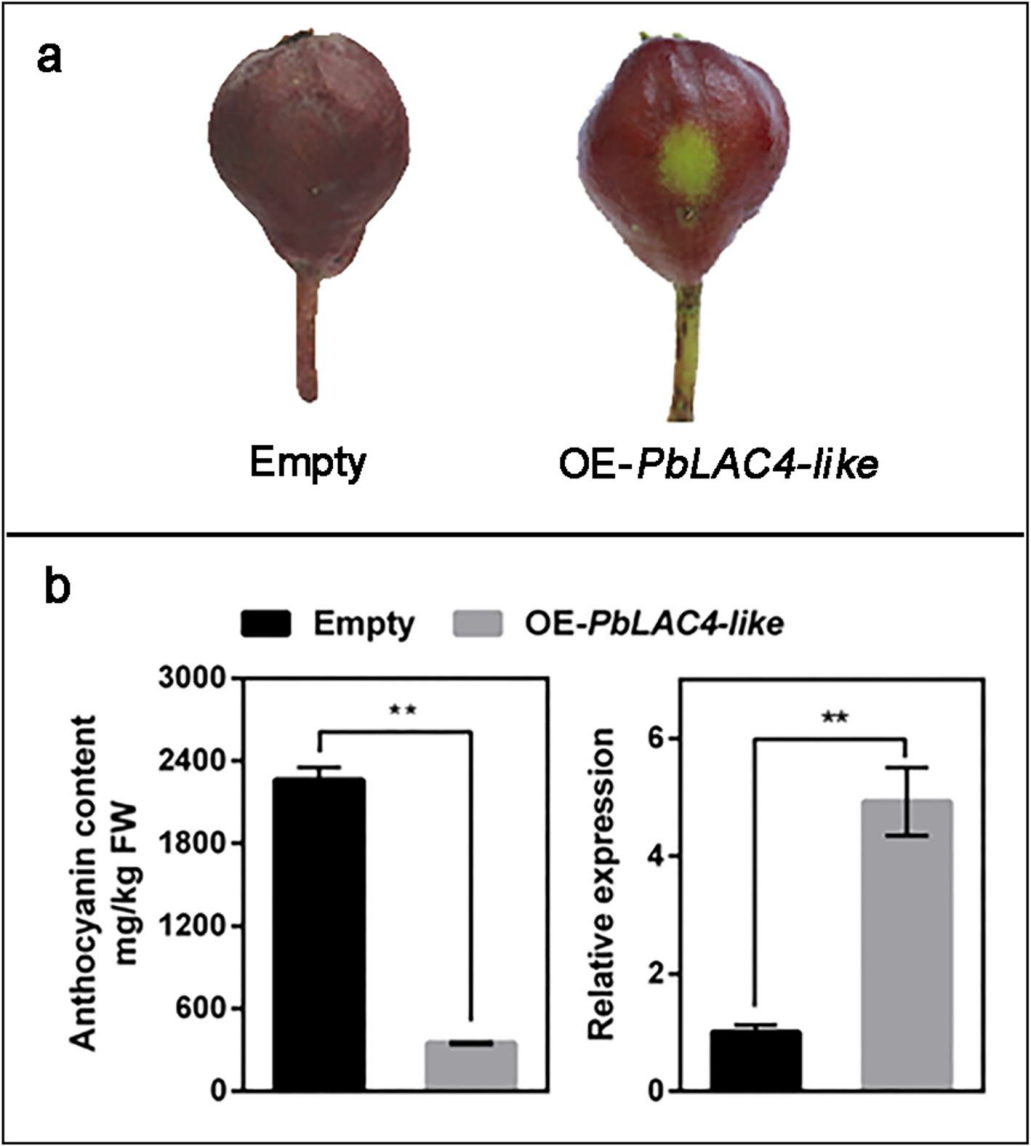
Transient transformation assays in the ‘Palacer’ fruitlet peel to verify the function of *PbLAC4-like*. **a** The phenotype of overexpressing *PbLAC4-like* (OE-*PbLAC4-like*) in ‘Palacer’ fruit, taking empty vector containing *GUS* (Empty) as the control. **b** Anthocyanin content and the expression level of *PbLAC4-like* gene in OE-*PbLAC4-like* and Empty. The significant difference was determined by t test for three replicates: *P < 0.05; **P < 0.01. Error bars represent the means ± SEM of three biological replicates

To verify the function of *PbLAC4-like* in pear, *PbLAC4-like* was transiently overexpressed in ‘Palacer’ fruitlet peel with faded color after bagging, taking the empty vector containing *GUS* as the control. GUS staining of the control showed that this transformation method was feasible (Additional file 2: Figure S2). After 10 days of transient transformation, the control returned to red, but the ‘Palacer’ fruitlet peel of overexpressed *PbLAC4-like* remained unstained (Fig. 4a). Compared with the control, anthocyanin content in overexpressed *PbLAC4-like* pear fruitlet peel decreased significantly, which was consistent with the observed phenotype (Fig. 4b). The expression level of *PbLAC4-like* in the overexpressed *PbLAC4-like* pear fruitlet peel was about five times higher than that in the control group, with a significant difference (Fig. 4b). In addition, we measured the expression levels of *PbMYB10* and *PbUFGT* in pericarp after transient overexpression of *PbLAC4-like*, and found that *PbMYB10* and *PbUFGT* were still expressed in this sample (Additional file 3: Figure S3), but the content of anthocyanin was reduced, indicating that overexpressing *PbLAC4-like* caused a decrease of anthocyanin content in pear fruitlet peel. Together, these results indicated that *PbLAC4-like* played a major role in the degradation of anthocyanin in pear.

### Detection of degrading anthocyanin activity of the PbLAC4-like protein

**Fig. 5:**
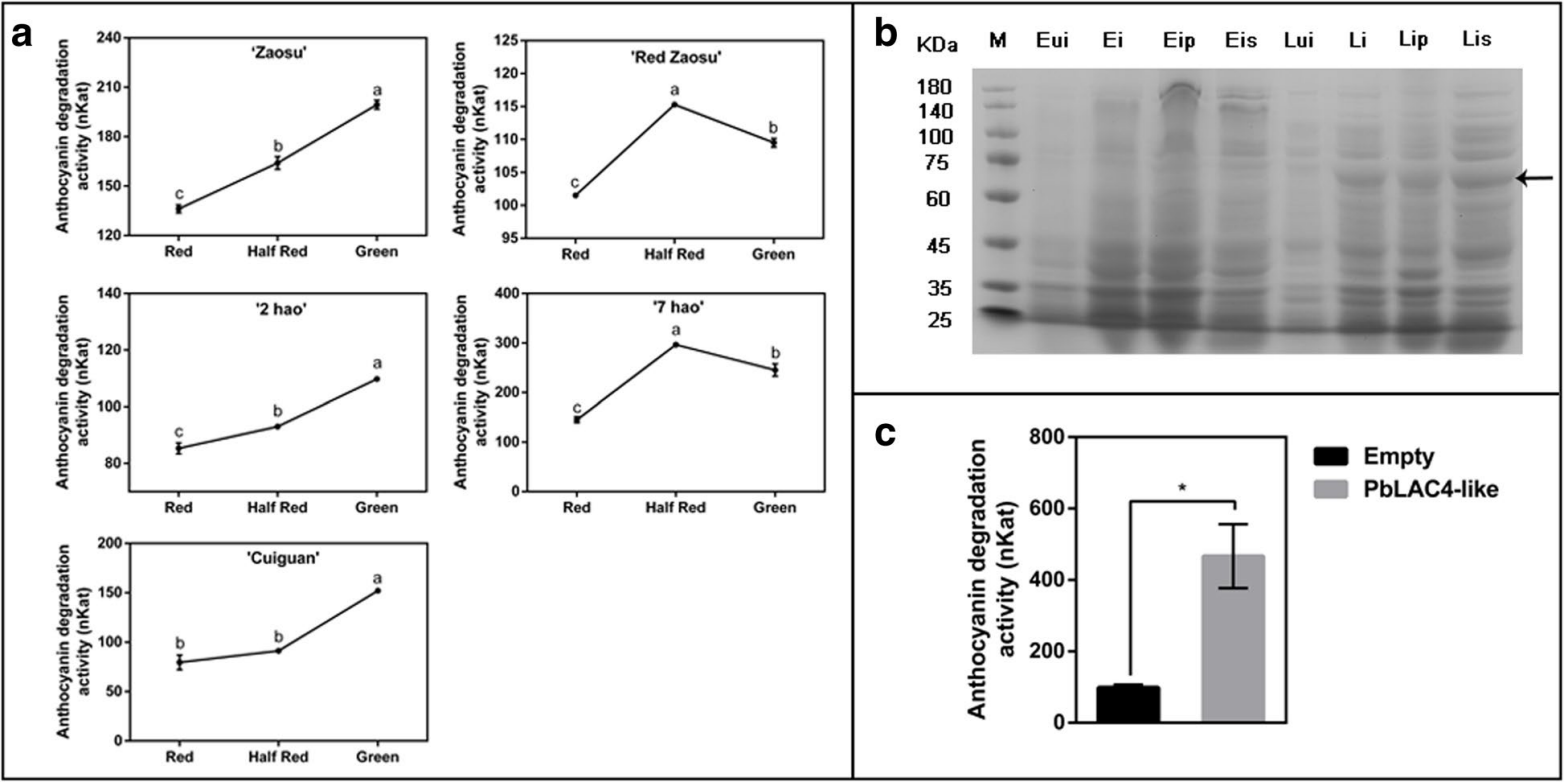
Anthocyanin degradation activity assay. **a** The activity assay of the crude enzyme to degrade anthocyanin in leaves of various varieties was carried out with anthocyanin as a substrate. **b** The SDS-PAGE gel of prokaryotic expression PbLAC4-like protein (M stood for the marker, Eui was pET28a (+) empty vector without induction, Ei was induced pET28a (+) empty vector, Eip and Eis were precipitation and supernatant of induced pET28a empty vector respectively, Lui stood for pET28a (+) -*PbLAC4-lik*e vector without induction, Li was induced pET28a (+) -*PbLAC4-lik*e vector and Lip and Lis was precipitation and supernatant of induced pET28a (+) -*PbLAC4-like* vector respectively). **c** The enzyme activity test of prokaryotic expressed PbLAC4-like protein was carried out with anthocyanin as a substrate. The significant difference was determined by t test for three replicates: *P < 0.05; **P < 0.01. Error bars represent the means ± SEM of three biological replicates

The anthocyanin degradation activity of crude enzyme of ‘Zaosu’, ‘Red Zaosu’, ‘2 hao’, ‘7 hao’ and ‘Cuiguan’ leaves were determined with anthocyanin as substrate. In ‘Zaosu’, ‘Red Zaosu’, ‘2 hao’ and ‘7 hao’ leaves, the activity of crude enzyme to degrade anthocyanin in Green and Half Red leaves was higher than that in Red leaves. In ‘Cuiguan’ leaves, the anthocyanin degradation activity of crude enzyme of Green leaves was higher than that of Half Red and Red leaves (Fig. 5a). The activity of crude enzyme to degrade anthocyanin was basically consistent with the *PbLAC4-like* expression level in leaves at different stages, the correlation values were shown in Table 4. In addition, PbLAC4-like protein was induced by prokaryotic expression. The size of PbLAC4-like protein was predicted by the protein molecular calculator, which was 60.9 kDa. In addition, there were two 6 × his tags on the pET28a (+) vector, each 6 × his tag was 840.9 Da, so the size of the fusion vector after prokaryotic expression was about 62.6 kDa. The sodium dodecyl sulfate polyacrylamide gel electrophoresis (SDS-PAGE) results showed that there was a band in the size of the his-PbLAC4-like fusion protein, indicating that the protein was successfully induced (Fig. 5b). The anthocyanin degradation activity of induced PbLAC4-like protein was measured by using anthocyanin as the substrate and induced empty vector protein as the control. The results showed that the induced PbLAC4-like protein activity was five times as that of the control group (Fig. 5c). In summary, at the protein level, the PbLAC4-like protein has been proved to be involved in the degradation of anthocyanin together with other anthocyanin degradation enzymes, and it was an important enzyme in the color fading process of pear leaves, petals and receptacles.

**Table 4:** Correlation analysis of anthocyanin content and anthocyanin degradation activity of crude enzyme in leaves

	Anthocyanin content				
	‘Zaosu’	‘Red Zaosu’	‘2 hao’	‘7 hao’	‘Cuiguan’
Anthocyanin degradation activity	−0.822**	−0.839**	−0.892**	− 0.860**	−0.702**

**Correlation is significant at the level of 0.01 (bilateral)

### Upstream transcriptional regulation of *PbLAC4-like* gene

**Fig. 6:**
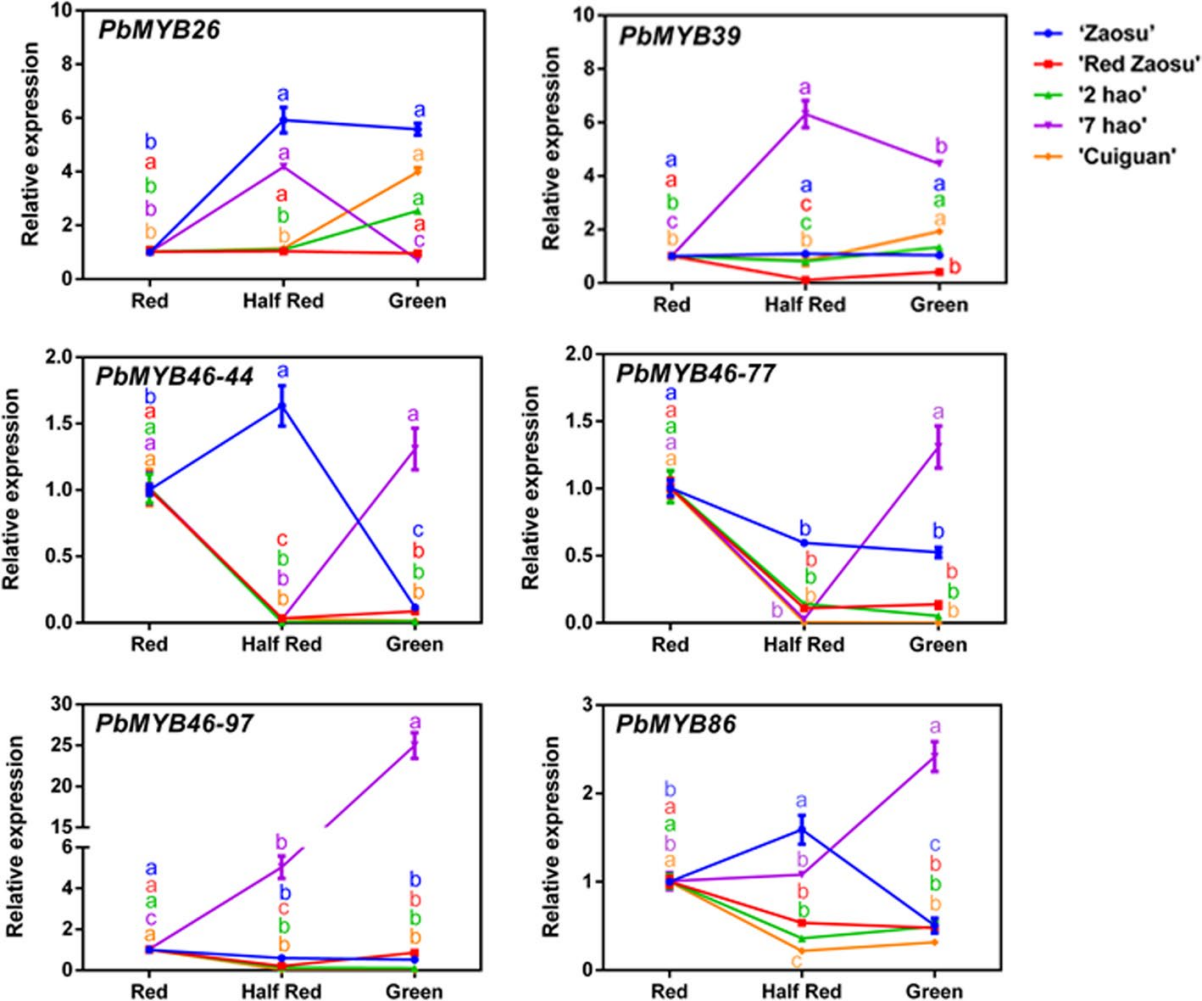
Expression analysis of candidate upstream regulators in pear leaves. The significant difference was determined by Tukey test for three replicates

**Fig. 7:**
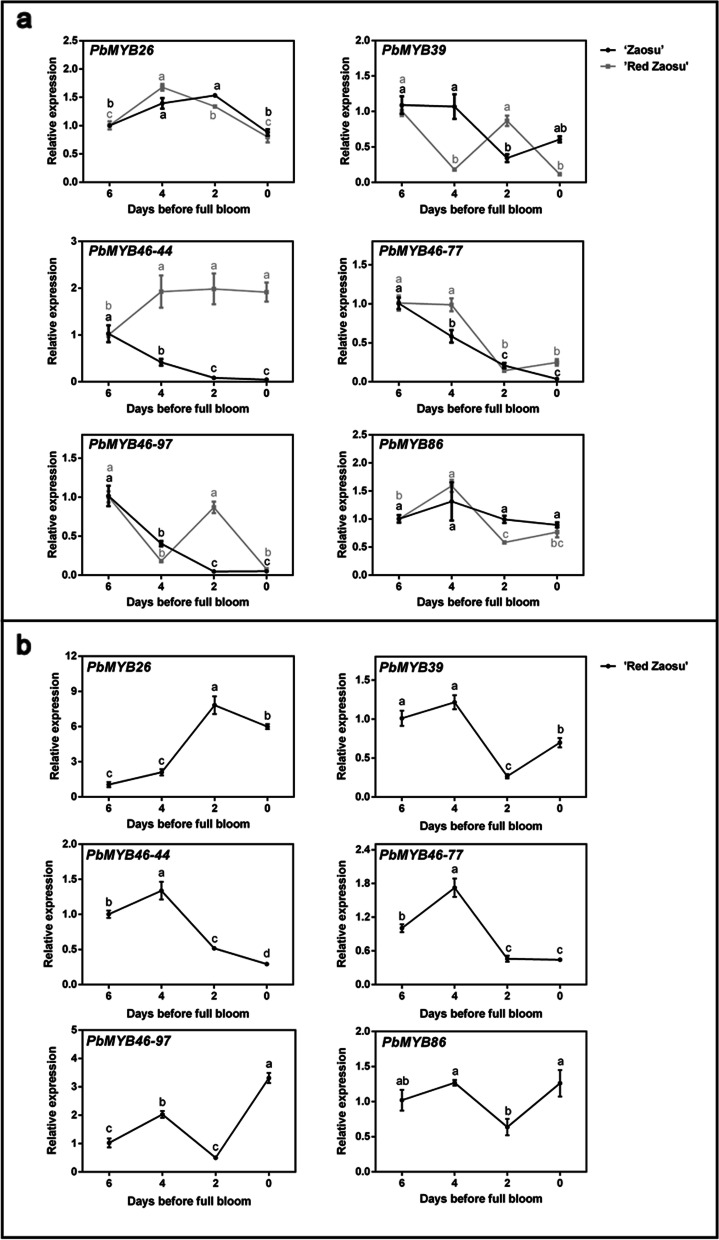
Expression analysis of candidate upstream regulators in petals and receptacles. **a** Expression levels of candidate upstream regulators in ‘Zaosu’, ‘Red Zaosu’ petals. **b** Expression levels of candidate upstream regulators in ‘Red Zaosu’ receptacles. The significant difference was determined by Tukey test for three replicates

**Fig. 8:**
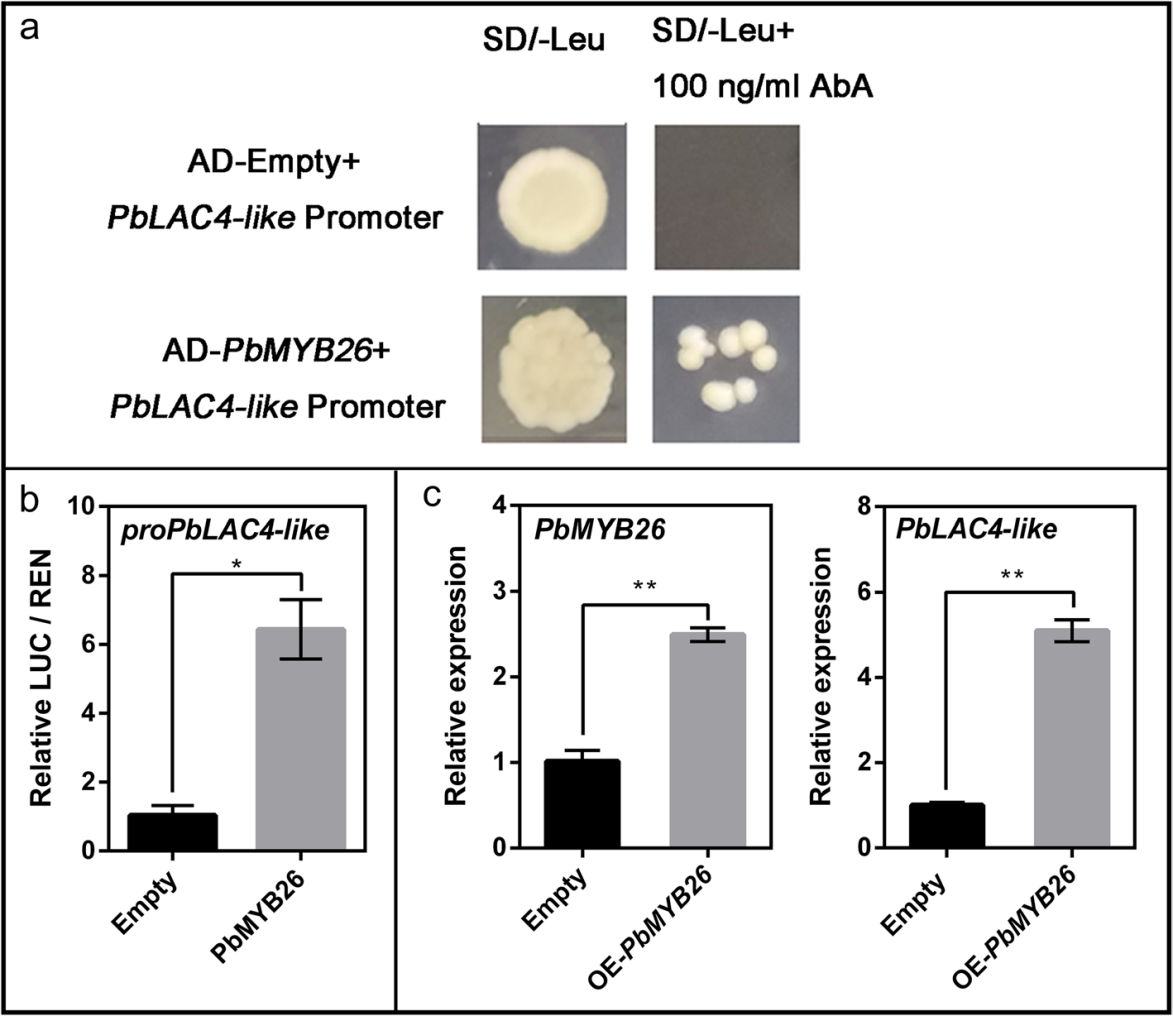
Validation of upstream regulators for the *PbLAC4-like* gene. **a** Y1H assay showing the interaction between *PbMYB26* and the *PbLAC4-like*. **b** Effects of *PbMYB26* on the promoter activity of *PbLAC4-like* in a dual luciferase assay. **c** Gene expression analysis after transient transformation *PbMYB26* gene in ‘Zaosu’ fruit peel. The significant difference was determined by t test for three replicates: *P < 0.05; **P < 0.01. Error bars represent the means ± SEM of biological replicates

*MYB46* was verified to positively regulate the expression of *LAC* genes by binding to the AC elements in their promoter regions in *Arabidopsis thaliana* [48]. In the present study, the AC elements were also identified in the promoter region of *PbLAC4-like*. So the proteins similar to AtMYB46 in pear genome were screened using phylogenetic analysis. PbMYB26, PbMYB39, PbMYB46–44, PbMYB46–77, PbMYB46–97 and PbMYB86 showed high homologies with AtMYB46 (Additional file 4: Figure S4). To further screen out the candidate regulators of *PbLAC4-like*, the expression patterns of these MYB genes were verified in leaves (‘Zaosu’, ‘Red Zaosu’, ‘2 hao’, ‘7 hao’ and ‘Cuiguan’), petals (‘Zaosu’, ‘Red Zaosu’), receptacles (‘Red Zaosu’). The results showed that only the expression pattern of *PbMYB26* was basically consistent with the expression pattern of *PbLAC4-like* in leaves, petals and receptals, showing an upward trend (Figs. 6, 7), which was also proved by the correlation values of *PbLAC4-like* and MYB candidates in leaves, petals, receptacles (Table 5). These results suggested that *PbMYB26* may take participate in regulating *PbLAC4-like e*xpression in pear. To confirm the possible interaction between PbLAC4-like and PbMYB26, yeast one-hybrid (Y1H) and dual luciferase assay were further conducted. The Y1H results showed that *PbMYB26* could directly bind to the *PbLAC4-like* promoter (Fig. 8a)*.* The effect of *PbMYB26* on the transcriptional activity of the *PbLAC4-like* promoter was determined by the relative LUC / REN ratio. Compared to the control, the relative LUC / REN of transient expressing *PbMYB26* was upregulated sixfold, indicating that *PbMYB26* upregulated the expression of *PbLAC4-like* (Fig. 8b)*.* Furthermore, the expression of *PbLAC4-like* was up-regulated in ‘Zaosu’ pear fruit with transient overexpression of *PbMYB26* (Fig. 8c). These results confirmed that *PbMYB26* activated *PbLAC4-like* expression by directly binding to the *PbLAC4-like* promoter.

**Table 5:** Correlation analysis of candidate transcription factors and *PbLAC4-like* expression levels in leaves, petals, receptacles

	*PbLAC4-like*		
	leaves	petals	receptacles
*PbMYB26*	0.741**	0.481*	0.924**
*PbMYB39*	−0.064	− 0.347	−0.836**
*PbMYB46–44*	−0.027	− 0.339	−0.567
*PbMYB46–77*	−0.136	− 0.670**	−0.559
*PbMYB46–97*	0.008	−0.562**	−0.296
*PbMYB86*	0.063	−0.049	−0.500

**Correlation is significant at the level of 0.01 (bilateral)

*Correlation is significant at the 0.05 level (bilateral)

## Discussion

Anthocyanin is a kind of plant pigment that can attract pollinators and promote plant pollination [6]. It can also help plants adapt to the environment in response to stresses [7–9]. The content of anthocyanin is affected by the biosynthesis and degradation of anthocyanin [27]. At present, the pathway of anthocyanin biosynthesis in pear is relatively clear. However, the degradation mechanism of anthocyanin has not been fully studied. In this study, an enzyme involved in the color fading of pear leaves, petals and receptacles was identified and characterized.

The *PbLAC4-like* gene was screened based on previous studies on the differences between ‘Zaosu’ and ‘Red Zaosu’ leaves [49], so the *PbLAC4-like* potentially involved in the accumulation of anthocyanin. In the later stage of color fading, the expression of *PbMYB10* and *PbUFGT* genes involved in anthocyanin biosynthesis decreased, but there was still a small amount of expression (Additional file 1: Figure S1), indicating that a small amount of anthocyanin could still be biosynthesized, but the content of anthocyanin decreased at this time (Figs. 1, 2), indicating that a large amount of anthocyanin was degraded. There was a negative correlation between the *PbLAC4-like* expression level and the anthocyanin content during the color fading of pear leaves, petals and receptacles (Figs. 1, 2 and Tables 1, 2, 3). So we hypothesized that the *PbLAC4-like* promoted the degradation of anthocyanin and caused redness continuously lost in pear.

The phylogenetic analysis in this study revealed that PbLAC4-like protein had high homologies with LAC in *Litchi chinensis* and LAC4, LAC11, LAC15 in *Arabidopsis thaliana* (Fig. 3). In *Litchi* fruit pericarp, LAC was responsible for epicatechin-mediated anthocyanin degradation [27]. In *Arabidopsis thaliana*, LAC15 involved in lignin and proanthocyanidin biosynthesis by oxidizing their respective monomers [50, 51]. LAC11 and LAC4 have been shown to play a role in lignin polymerization in *Arabidopsis thaliana* [39, 41, 52]. The *PbLAC4-like* was screened out from the different genes between ‘Zaosu’ and ‘Red Zaosu’ leaves [49], so we speculated that the PbLAC4-like protein involved in the degradation of anthocyanin. Sequence alignment revealed that PbLAC4-like protein, LAC in *Litchi chinensis* and *Arabidopsis thaliana* had three Cu-oxidase domains that could oxidize substrates [53]. The structure indicated that PbLAC4-like protein probably participated in the degradation of anthocyanin by oxidizing anthocyanin.

The color fading of ‘Red Bartlett’ fruit might be related to LAC [34]. LAC involved in the degradation of anthocyanin during *Litchi* fruit pericarp browning [27]. LAC15 involved in the oxidation of flavonoids resulting in *Arabidopsis thaliana* seed coat browning [50]. In the present study, the function of the PbLAC4-like protein to degrade anthocyanin in pear was demonstrated. The content of anthocyanin in ‘Palacer’ fruitlet peel after overexpressing *PbLAC4-like* was less than that in control (Fig. 4). The activity to degrade anthocyanin of induced PbLAC4-like protein was significantly higher than that of control (Fig. 5). And we found that the color fading of pear leaves, petals, receptacles were all related to *PbLAC4-like*. However, in this study, PbLAC4-like protein directly degraded anthocyanin and did not need other substances to assist. This was different from the degradation of anthocyanin in *Litchi* fruit pericarp caused by LAC required the presence of epicatechin, and LAC played a role in anthocyanin degradation based on the oxidation of epicatechin [27]. In addition, which anthocyanin group did the PbLAC4-like protein act on and what were the degraded products were still unclear and need further study.

So far, there have been many studies on the regulators of *LAC*. *MYB26, MYB46* and *MYB83* in *Arabidopsis thaliana* [45–48], *miR397a* and *MYB169* in pear [43, 44] regulated the expression of *LAC*. All of these studies were that the transcription factors involved in lignin accumulation by regulating *LAC*. However, there was no report that transcription factors involved in anthocyanin degradation by regulating LAC. Here, the expression pattern of *MYB*26 was consistent with that of *PbLAC4-like* in pear leaves, petals and receptacles (Figs. 6, 7). Y1H and dual luciferase assay showed that *PbMYB26* could directly bind to the *PbLAC4-like* promoter and activated its transcription, and this result was also verified by transient expression in pear (Fig. 8). However, whether *PbMYB26* regulated *PbLAC4-like* by binding to the AC element in the *PbLAC4-like* promoter region remained to be further studied. Taken together, we hypothesized that *PbMYB26* might involve in the degradation of anthocyanin by activating the *PbLAC4-like* promoter. These findings provide new ideas for further research on transcription factors that regulate the degradation of anthocyanin.

## Conclusion

The biosynthesis and degradation of anthocyanin affect the content of anthocyanin. There have been many reports on the mechanism of anthocyanin biosynthesis, but the degradation mechanism of anthocyanin has not been fully studied. In this study, in the early development of pear leaves, petals and receptacles, anthocyanin is biosynthesized more and degraded less, so the accumulation of anthocyanin makes the leaves, petals and receptacles appear red. During the red color fading of pear leaves, petals and receptacles, the accumulation of anthocyanin decreased, but a small amount of anthocyanin was still biosynthesized, indicating that the decrease in anthocyanin content at this time was caused by the massive degradation of anthocyanin. The effect of *PbLAC4-like* on the degradation of anthocyanin in pear was verified by transient transformation and anthocyanin degradation activity determination. The upstream transcriptional regulators of *PbLAC4-like* have been preliminarily explored. *PbMYB26* might relate to the degradation of anthocyanin by directly activating the *PbLAC4-like* promoter. Taken these results together, *PbLAC4-like* played an important role in the color fading process of pear leaves, petals and receptacles. Studying the degradation mechanism of anthocyanin can not only better understand the color fading process in pear, but also provide a new perspective for inhibiting the degradation of anthocyanin and promoting the accumulation of anthocyanin.

## Methods

### Plant materials and treatment methods

In this study, pear leaves, petals and receptacles were collected from the Horticultural Research Base of Northwest A&F University in Yangling, Shaanxi, China. Due to the difference in the time it takes for each variety’s leaf color to fade, we classified the leaves into Red, Half Red and Green according to their color phenotypic characteristics. The Red, Half Red and Green leaves of ‘Zaosu’ (*Pyrus bretschneideri* Rehd.), ‘Red Zaosu’ (*Pyrus bretschneideri* Rehd.), ‘2 hao’ (Inter specific *Pyrus* hybrid), ‘7 hao’ (Inter specific *Pyrus* hybrid), ‘Cuiguan’ (*Pyrus pyrifolia* Nakai.) were collected from the upper, middle and lower phyllotaxis of their branches respectively on the same day. ‘Zaosu’, ‘Red Zaosu’ petals and ‘Red Zaosu’ receptacles were collected at 6 days, 4 days, 2 days and 0 days before full bloom, respectively. All samples were collected and immediately frozen in liquid nitrogen and stored at − 80 °C. Moreover, ‘Palacer’ (*Pyrus communis* L.) and ‘Zaosu’ fruitlets grown in Meixian, Shaanxi, China, were selected for transient transformation to verify the function of *PbLAC4-like* and *PbMYB26*. *Nicotiana benthamiana* seedlings that had six leaves were used for dual luciferase activity assay.

### The details of plant materials

‘Zaosu’ was identified formally by the Institute of Fruit Science, Chinese Academy of Agricultural Sciences. ‘Red Zaosu’, ‘2 hao’ and ‘7 hao’ were identified formally by our lab, while ‘Red Zaosu’ was a spontaneous bud sport of the ‘Zaosu’, ‘2 hao’ and ‘7 hao’ were the hybrid offspring of *Pyrus pyrifolia*, ‘Cuiguan’ was identified formally by Zhejiang Academy of Agricultural Sciences. These materials have been deposited in a publicly available herbarium. They were stored in the Pear Variety Resource Nursery of Northwest A&F University. The permissions were obtained to cultivate all the plants used in the study.

### DNA and RNA extraction and purification

The total RNA was extracted using an RNAprep Pure Plant Kit (TIANGEN, Beijing, China) in accordance with the manufacturer’s instructions. The RNA concentration and quality were tested using Multiskan GO (Thermo, MA, USA). The first-strand cDNA was synthesized from 1 μg of total RNA using a PrimeScript RT reagent kit with gDNA Eraser (TaKaRa, Dalian, China).

### Sequence analysis of LAC

The protein sequences of LAC from *Arabidopsis thaliana*, *Litchi chinensis*, and *Pyrus bretschneideri* were used to construct a phylogenetic tree in the MEGA 5.0 software by Neighbor–Joining method and JTT + G model. Bootstrap values were calculated from 1000 replicate analyses. The protein sequences were aligned using DNAMAN. The GenBank accessions of related protein are listed in Additional file 5: Table S1.

### Expression analysis using quantitative real-time PCR (qRT-PCR)

The primers for selected genes and *PbActin* (an internal control) were designed on NCBI web pages and were synthesized by AuGCT Biotech Company (Beijing, China). The primers are listed in Additional file 6: Table S2. The qRT-PCR reactions were performed on an Applied Biosystems StepOnePlus™ Real-Time PCR Systems (Applied Biosystems, Waltham, MA, USA) with TB Green Premix Ex Taq II (Tli RNaseH Plus; TaKaRa, Dalian, China) according to the manufacturer’s instructions. Transcript levels of three biological replicates were analyzed using the cycle threshold (2^−ΔΔCt^) method.

### Determination of the anthocyanin content

The extraction and quantification of anthocyanin were performed using a previously reported method, with slight modifications [54]. The anthocyanin was extracted using homogenizing method with polyphenol extracting solution, which consisted of 50% methanol, 48% water and 2% formic acid at 4 °C. The supernatant was filtered by 0.22 μm organic filter for the determination of anthocyanin by high performance liquid chromatography (HPLC). Anthocyanin was analyzed using a LC-20A Liquid Chromatograph equipped with a diode array detector (Shimadzu Corporation, Tokyo, Japan). An Inertsil ODS-3 column (5.0 μm, 4.6 × 250 mm, GL Sciences Inc., Tokyo, Japan) was used in the separation. Solvent A consisted of 10% formic acid (HPLC grade, purity: 88%) and 90% water, and solvent B was 10% formic acid (HPLC grade, purity: 88%) dissolved in acetonitrile (HPLC grade, purity: 99.9%). The gradient elution procedure was 92% solvent A (0 min), 60% solvent A (10 min), 92% solvent A (24 min). The flow rate was 1.0 mL min^− 1^ at 30 °C. Simultaneous monitoring was performed at 520 nm for anthocyanin (cyanidin-3-galactoside). Peaks were identified by a comparison of retention times and UV spectra with anthocyanin standard. The concentrations of anthocyanin in three biological replicates were determined according to the peak areas and calibration curves that were made with different concentrations of anthocyanin standard. The standard was obtained from Yuanye Bio-Technology (Shanghai, China).

### Transient overexpression assay in pear fruitlet peel

The transient overexpression method referred to previous studies, and the fruitlets after transient overexpression grow in the sun [16, 17]. The complete coding sequences (CDS) of *PbLAC4-like* and *PbMYB26* were amplified by PCR from ‘Red Zaosu’ cDNA and then fused into the multiple cloning site (MCS) of pGreenII 0029-62SK vector respectively to form 62SK-*PbLAC4-like* and 62SK-*PbMYB26* plasmids. The CDS of *GUS* was PCR-amplified from pBI121 and then inserted into the MCS of pGreenII 0029-62SK vector to form 62SK-*GUS* plasmid that as a control Empty. The primers for amplifying the sequences are described in Additional file 7: Table S3. The infusion vectors were transferred into *Agrobacterium tumefaciens* strain EHA105, and incubated in Luria-Bertani medium. After the activation of *Agrobacterium*, they were suspended with resuspension (10 μmol/L MES, 10 μmol/L MgCl_2_, pH = 5.6, and 200 μmol/L AS) and cultured in dark for 3 h at room temperature, and then the OD_600_ was adjusted to 0.6. The 62SK-*GUS* and 62SK-*PbLAC4-like* bacterial solutions were injected into ‘Palacer’ fruitlet peel with faded color after bagging. The 62SK-*GUS* and 62SK-*PbMYB26* bacterial solutions were injected into ‘Zausu’ fruitlet peel. Each treatment consisted of 3 biological replicates and each biological replicate contained 5 fruitlets. Three days after injection, pericarp at the injection site was collected for quantitative analysis, and the phenotypic was analyzed 10 days after injection. 62SK-*GUS* can be accelerated by 35S promoter, that is, the transcription of GUS could be accelerated by 35S promoter. To prove the feasibility of this transformation method, GUS staining was performed on the transient *GUS* sites according to the previous method [55].

### Crude enzyme extraction and anthocyanin degradation activity assay

The crude enzyme was extracted by homogenizing the powder with polyvinylpyrrolidone (10% of the leaf by weight) and 2 mL extracting solution which consisted of 0.02 M citrate buffer (pH 5.0), 0.02 M anhydrous calcium chloride, 0.005 M Dithiothreitol, 0.01 M thiourea. The homogenate was centrifuged for 20 min at 12,000 rpm and 4 °C, and then the supernatant was collected after passing the PD-10 desalting column (GE Healthcare) as the crude enzyme.

The anthocyanin degradation activity assay was performed according to the method of Zhang et al. [30] with minor changes. The enzyme was added to the 200 μL 0.2 M citrate buffer (pH 4.0) with 0.2 mM anthocyanin standard sample. The mixture was incubated for 30 min at 42 °C. The reaction was terminated with 0.1 M hydrochloric methanol solution. The content of anthocyanin was measured by HPLC. The PbLAC4-like protein activity was expressed as the degradation of 1 μmol anthocyanin per minute at 42 °C.

### Prokaryotic expression of the *PbLAC4-like*

Prokaryotic expression of the *PbLAC4-like* was carried out according to the method of Kampatsikas et al. [56] with slight modifications. The CDS of *PbLAC4-like* was PCR-amplified and cloned into the pET-28a (+) vector. The primers are listed in Additional file 7: Table S3. The resulting construct was transformed into *Escherichia coli* strain BL21 (DE3). The *E. coli* BL21 (DE3) was cultured in 2 × YT medium with ampicillin (100 μg/ml) at 37 °C until its OD_600_ value was 0.6. Then 0.5 mM isopropyl β-D-1-thiogalactopyranoside and 0.5 mM CuSO_4_ were added for protein induction. The expression culture was shaken for 8 h at 28 °C, centrifuged at 4 °C, and the supernatant was discarded. The precipitation was suspended with 0.02 M citrate buffer (pH 5.0) for ultrasonic crushing. The broken expression culture was centrifuged at 12000 rpm for 30 min at 4 °C, and the supernatant was taken for enzyme activity assay.

### Yeast one-hybrid assay

The *PbLAC4-like* promoter sequence was cloned into the bait vector pAbAi, and the CDS of *PbMYB26* was inserted into the prey vector pGADT7. The primers used to amplify the *PbLAC4-like* promoter and the CDS of *PbMYB26* are listed in Additional file 7: Table S3. The pAbAi-bait vector was digested by BbsI and transferred into yeast strain Y1HGOLD to construct the bait yeast strain. To determine the minimum aureobasidin A (AbA) inhibitory concentration of the bait yeast strain, the bait yeast strain was cultured on the SD/−Ura medium containing 50–200 ng ml^− 1^ AbA. Then, transferring the prey plasmid into the bait yeast strain and culturing it on the SD/−Leu medium containing the minimum AbA inhibitory concentration that was screened out before to verify the interaction.

### Dual luciferase assay in Nicotiana benthamiana leaves

To assay the effect of *PbMYB26* on *PbLAC4-like*, the promoter of *PbLAC4-like* was amplified and inserted into the MCS of pGreenII 0800-LUC double-reporter plasmid as reporter. The effector plasmid was constructed by inserting the CDS of *PbMYB26* into the MCS of pGreenII 0029-62SK vector. The related primers are listed in Additional file 7: Table S3. *Agrobacterium tumefaciens* containing reporter plasmid and effector plasmid was injected into *Nicotiana benthamiana* leaves at a ratio of 1 to 4. The empty pGreenII 0029-62SK plasmid and pGreenII 0800-LUC-pro*PbLAC4-like* plasmid were injected into *Nicotiana benthamiana* leaves in the same proportion as control. After injection, *Nicotiana benthamiana* was cultured in dark for 12 h and then cultured under the light. The activity of LUC and REN was measured 3 days after injection using a dual LUC assay kit (Promega, Madison, WI, USA) and an Infinite M200pro Full Wavelength Multifunctional Enzyme Standard Instrument (TECAN, Männedorf, Switzerland). Five biological repeats were included for each treatment.

### Statistical analysis

Statistical analysis was performed using SPSS 20 software (SPSS, Chicago, IL, USA). Tukey test was conducted to determine the significant difference between three or more samples, and t test was used to detect the significant difference between two samples. The correlation analysis among the date was carried out by SPSS 20 software. Each value represents the mean ± SEM of three biological replicates. Figures were made using GraphPad Prism 6 software (GraphPad Prism, San Diego, CA, USA).

## Supplementary Information

**Additional file 1: Figure S1.** Expression level of *PbMYB10* and *PbUFGT* in pear leaves, petals and receptacles. The significant difference was determined by Tukey test for three replicates.

**Additional file 2: Figure S2.** GUS staining of the pear fruit after injecting pGreenII0029 62SK-*GUS*.

**Additional file 3: Figure S3.** Expression level of *PbMYB10* and *PbUFGT* in ‘Palacer’ fruit after transient overexpression of *PbLAC4-like*.

**Additional file 4: Figure S4.** Phylogenetic analysis of MYB46 in *Arabidopsis thaliana* and MYB transcription factors in pears.

**Additional file 5: Table S1.** The GenBank accessions of related proteins.

**Additional file 6: Table S2.** Sequences of primers used for qRT-PCR analysis.

**Additional file 7: Table S3.** Sequences of primers used for vector construction.

**Additional file 8.** Title page.

**Additional file 9.** The original gel of Fig. 5b.

**Additional file 10.** The first multiple exposure image of Fig. 5b.

**Additional file 11.** The second multiple exposure image of Fig. 5b.

**Additional file 12.** The third multiple exposure image of Fig. 5b.

## Data Availability

All data generated or analysed during this study are included in this published article and its supplementary information files. The materials are available upon request by contacting the corresponding author. The datasets generated during the current study are available in the GenBank repository, http://www.ncbi.nlm.nih.gov/ and the accession numbers are listed in Additional files 5 and 6.

## Abbreviations

UFGT: Flavonoid 3-O-glucosyl transferase
MBW: MYB-bHLH-WD40
POD: Peroxidases
PPO: Polyphenol oxidases
LAC: Laccases
CDS: Complete coding sequences
MCS: Multiple cloning site
AbA: Aureobasidin A
SDS-PAGE: Sodium dodecyl sulfate polyacrylamide gel electrophoresis
Y1H: Yeast one-hybrid
OE: Overexpression
S: Stage
PCR: Polymerase chain reaction
