# Supplementary figures and images for: PbLAC4-like, activated by PbMYB26, related to the degradation of anthocyanin during color fading in pear
Source: BMC Plant Biol. 2021 Oct 13;21:469. doi: 10.1186/s12870-021-03220-1 (PMC8515750; doi:10.1186/s12870-021-03220-1)

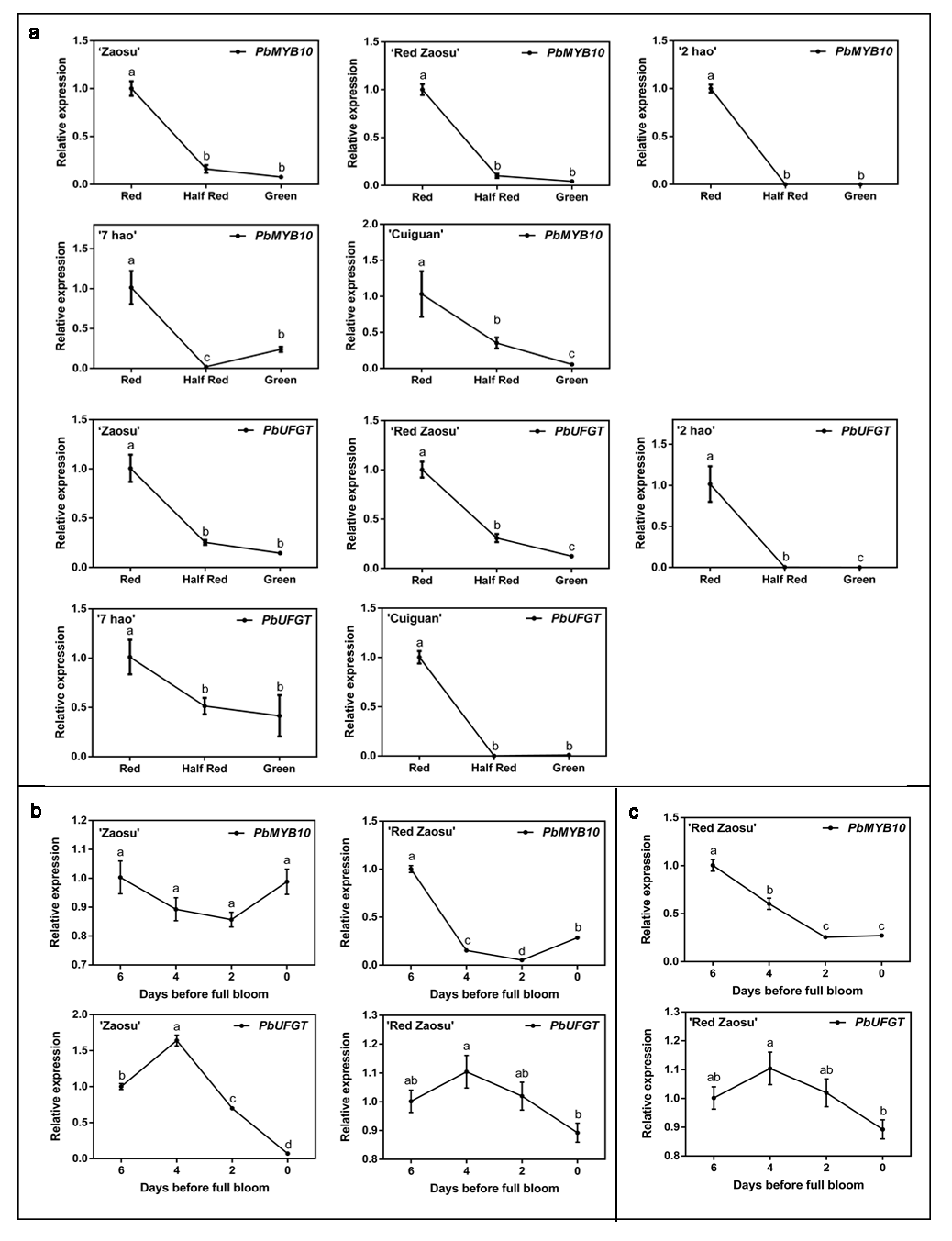

Supplement: Supplementary file 1 — Additional file 1: Figure S1. Expression level of PbMYB10 and PbUFGT in pear leaves, petals and receptacles. The significant difference was determined by Tukey test for three replicates. [file 12870_2021_3220_MOESM1_ESM.tif]

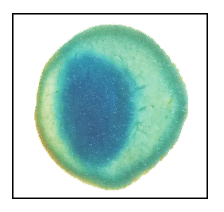

Supplement: Supplementary file 2 — Additional file 2: Figure S2. GUS staining of the pear fruit after injecting pGreenII0029 62SK-GUS. [file 12870_2021_3220_MOESM2_ESM.tif]

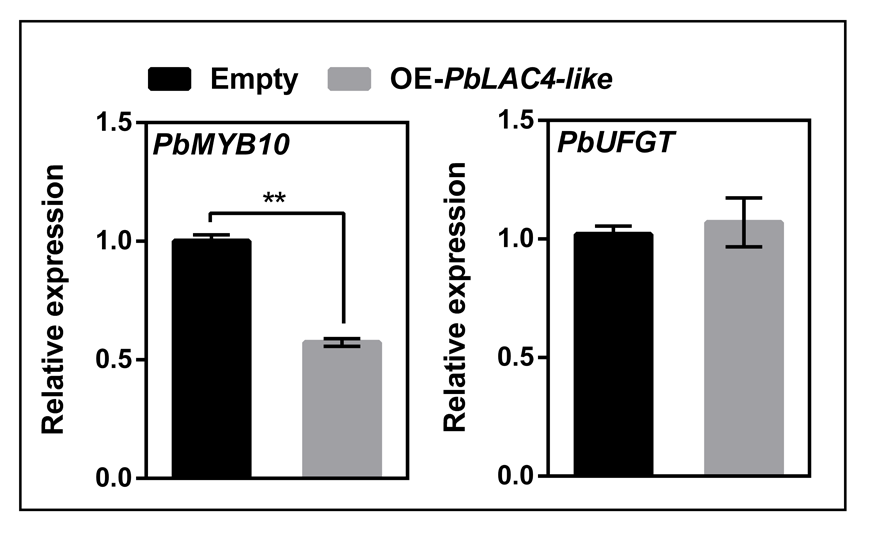

Supplement: Supplementary file 3 — Additional file 3: Figure S3. Expression level of PbMYB10 and PbUFGT in ‘Palacer’ fruit after transient overexpression of PbLAC4-like. [file 12870_2021_3220_MOESM3_ESM.tif]

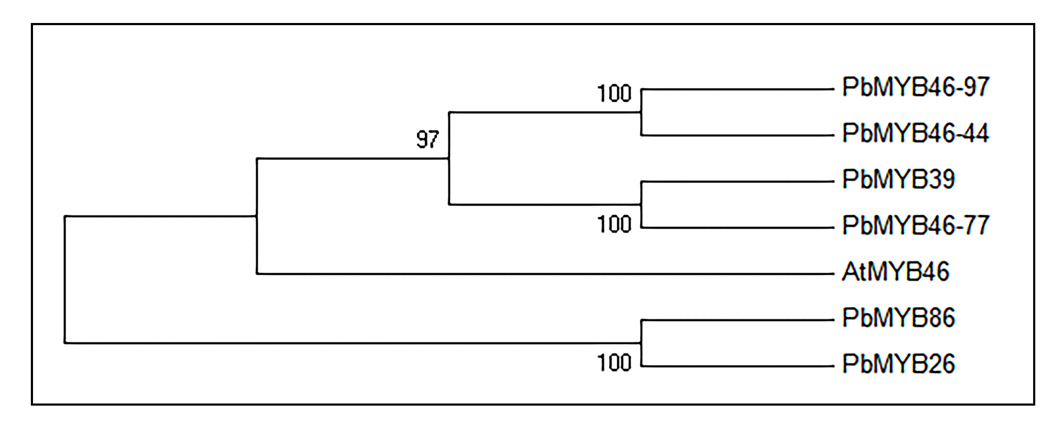

Supplement: Supplementary file 4 — Additional file 4: Figure S4. Phylogenetic analysis of MYB46 in Arabidopsis thaliana and MYB transcription factors in pears. [file 12870_2021_3220_MOESM4_ESM.tif]

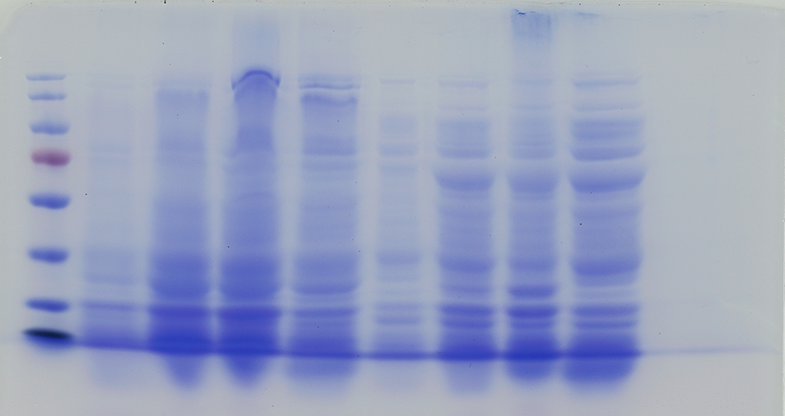

Supplement: Supplementary file 9 — Additional file 9. The original gel of Fig. 5b. [file 12870_2021_3220_MOESM9_ESM.tif]

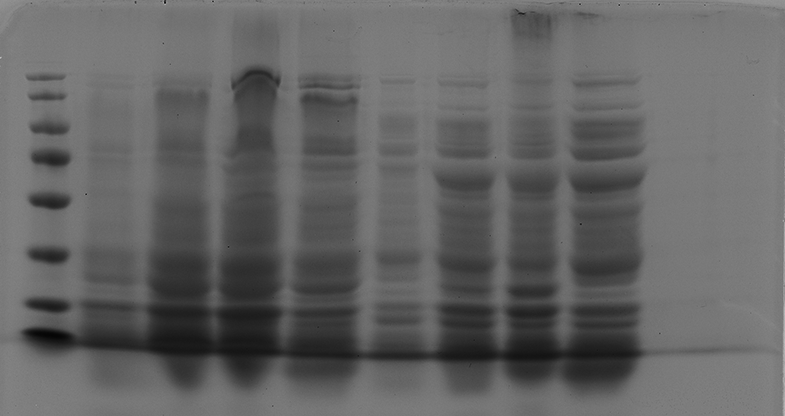

Supplement: Supplementary file 10 — Additional file 10. The first multiple exposure image of Fig. 5b. [file 12870_2021_3220_MOESM10_ESM.tif]

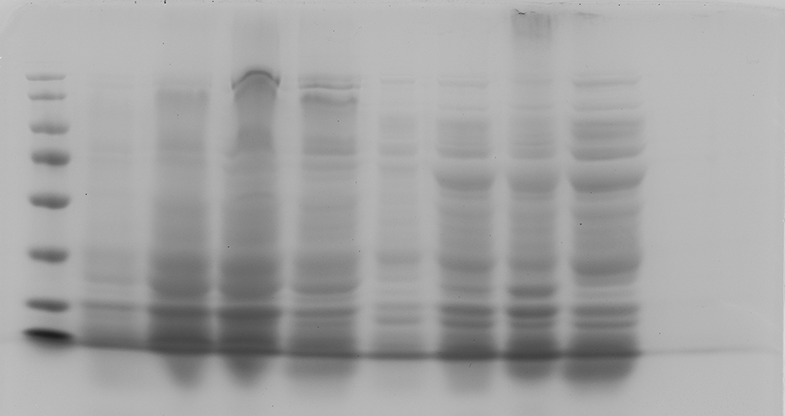

Supplement: Supplementary file 11 — Additional file 11. The second multiple exposure image of Fig. 5b. [file 12870_2021_3220_MOESM11_ESM.tif]

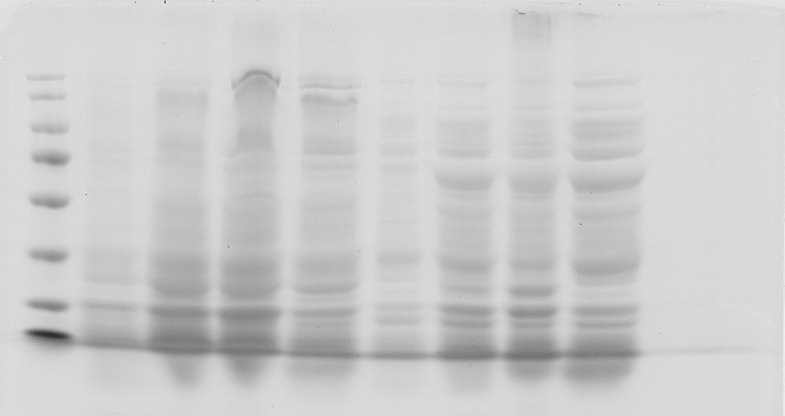

Supplement: Supplementary file 12 — Additional file 12. The third multiple exposure image of Fig. 5b. [file 12870_2021_3220_MOESM12_ESM.tif]
