# Supplementary material for: PbLAC4-like, activated by PbMYB26, related to the degradation of anthocyanin during color fading in pear
Source: BMC Plant Biol. 2021 Oct 13;21:469. doi: 10.1186/s12870-021-03220-1 (PMC8515750; doi:10.1186/s12870-021-03220-1)
Supplement: Supplementary file 8 — Additional file 8. Title page. [file 12870_2021_3220_MOESM8_ESM.docx]

**Title: *PbLAC4-like*, activated by *PbMYB26*, related to the degradation of anthocyanin during color fading in pear**

**Authors:** Guangping Zhao^1^, Fangxin Xiang^1^, Shichao Zhang^1^, Junxing Song^1^, Xieyu Li^1^, Linyan Song^1^, Rui Zhai^1^*, Chengquan Yang^1^, Zhigang Wang^1^, Fengwang Ma^1,2^, Lingfei Xu^1^*

^1^College of Horticulture, Northwest A&F University, Taicheng Road NO.3, Yangling, Shaanxi Province, China.

^2^State Key Laboratory of Crop Stress Biology for Arid Areas, Northwest A&F University, Taicheng Road NO.3, Yangling, Shaanxi Province, China.

**The email addresses:**

Guangping Zhao: [zhaogp1996@nwafu.edu.cn;](mailto:zhaogp1996@nwafu.edu.cn;)

Fangxin Xiang: [xiangfangxin@nwafu.edu.cn;](mailto:xiangfangxin@nwafu.edu.cn;)

Shichao Zhang: [woshizsc@outlook.com;](mailto:woshizsc@outlook.com;)

Junxing Song: [JunxingSong@163.com;](mailto:JunxingSong@163.com;)

Xieyu Li: [junyouki@sina.com;](mailto:junyouki@sina.com;)

Linyan Song: [linyans@yeah.net;](mailto:lingyans@yeah.net;)

Rui Zhai: [Zhai.Rui@nwafu.edu.cn](mailto:Zhai.Rui@nwafu.edu.cn)

Chengquan Yang: [cqyang@nwsuaf.edu.cn;](mailto:cqyang@nwsuaf.edu.cn;)

Zhigang Wang: [wzhg001@163.com;](mailto:wzhg001@163.com;)

Fengwang Ma: [fwm64@sina.com;](mailto:fwm64@sina.com;)

Lingfei Xu: [lingfxu2013@sina.com](mailto:lingfxu2013@sina.com).

**Institution: College of Horticulture, Northwest A&F University**

**Address of the institution: Taicheng Road NO.3, Yangling, Shaanxi Province, China**

*: Lingfei Xu and Rui Zhai are co-corresponding authors.

Lingfei Xu

College of Horticulture, Northwest A&F University, Taicheng Road NO.3, Yangling, Shaanxi Province, China.

E-mail: [lingfxu2013@sina.com](mailto:lingfxu2013@sina.com), Tel.: (86) 029-87081023

Rui Zhai

College of Horticulture, Northwest A&F University, Taicheng Road NO.3, Yangling, Shaanxi Province, China.

E-mail: Zhai.Rui@nwafu.edu.cn, Tel.: 86-029-13484475409
